# Testing assumptions for endophenotype studies in ADHD: Reliability and validity of tasks in a general population sample

**DOI:** 10.1186/1471-244X-5-40

**Published:** 2005-11-01

**Authors:** Jonna Kuntsi, Penny Andreou, Jonathan Ma, Norbert A Börger, Jaap J van der Meere

**Affiliations:** 1 MRC Social, Genetic and Developmental Psychiatry Centre, Institute of Psychiatry, King's College London, De Crespigny Park, London SE5 8AF, UK; 2 Department of Developmental and Experimental Clinical Psychology, University of Groningen, Grote Kruisstraat 2/1, 9712 TS Groningen, The Netherlands

## Abstract

**Background:**

Advances in both genetic and cognitive-experimental studies on attention deficit hyperactivity disorder (ADHD) have opened new opportunities for cognitive endophenotype research. In such genetic designs the focus is on individual differences in characteristics, associated with ADHD, that can be measured reliably over time. Genetic studies that take a 'quantitative trait loci' approach hypothesise that multiple susceptibility genes contribute to a continuous dimension of ADHD symptoms. As an important initial step, we aimed to investigate the underlying assumptions that (1) key cognitive-experimental tasks indicate adequate test-retest reliability and (2) ADHD symptom scores in a general population sample are associated with performance on these tasks.

**Methods:**

Forty-nine children were assessed on a go/no-go task and a reaction time task (the 'fast task') that included manipulations with event rate and incentives. The children were assessed twice, with a test-retest interval of two weeks.

**Results:**

The majority of the task variables demonstrated moderate-to-good test-retest reliability. The correlations between teacher ratings of ADHD symptoms and key task variables were .4–.6: ADHD symptoms were associated with poor performance (especially high reaction time variability) in a slow baseline condition, whereas there was low or no association in conditions with a faster event rate or incentives. In contrast, no clear pattern of findings emerged based on parent ratings of ADHD symptoms.

**Conclusion:**

The data support the usefulness of the go/no-go and fast tasks for genetic studies, which require reliable and valid indices of individual differences. The overall pattern of associations between teacher ratings of ADHD symptoms and task variables is consistent with effects of event rate and incentives on performance, as predicted by the model of activation and arousal regulation. The lack of a clear pattern of findings with parent ratings of ADHD symptoms warrants further study.

## Background

Attention deficit hyperactivity disorder (ADHD) has been the focus of numerous both genetic and cognitive-experimental studies in recent years. The symptom cluster of impulsivity, overactivity and inattentiveness has been shown to be highly heritable and molecular genetic studies have obtained initial evidence of genes that contribute to the disorder (reviewed in [1-3]). Cognitive-experimental studies have indicated tasks that are sensitive to ADHD, pointing to possible underlying psychological processes [4,5]. These advances have led to an increased interest in planning investigations on cognitive endophenotypes in ADHD [4]. Endophenotypes refer to quantifiable intermediate constructs that index an underlying liability to a disorder or behavioural trait. By studying candidate endophenotypes we can start to unravel the causal pathways from etiological factors through to psychological processes and behaviour.

Certain key assumptions underlie such an approach that combines genetic and cognitive-experimental methods. As the focus is on differences between individuals in etiological factors that contribute to the disorder, it is critical that the candidate endophenotypes reflect characteristics on which individuals differ *reliably *over time. Demonstrating adequate test-retest reliability has been acknowledged as an important initial step [6], but few studies have as yet obtained such data. Although an unusually high variability in performance is one of the strongest findings yet to emerge from cognitive-experimental research on ADHD [4,5,7], this needs not pose a problem for test-retest reliability: if variability in performance indicates a true underlying process, we can aim to obtain reliable indicators of this characteristic.

Whereas some genetic studies on ADHD follow a strict diagnostic (categorical) approach, others are based on an underlying QTL (quantitative trait loci) assumption of multiple susceptibility genes contributing to a continuous dimension of ADHD symptoms. Twin study data are consistent with the hypothesis that ADHD represents the extreme of a behaviour that varies continuously throughout the entire population [8,9]. From this hypothesis it follows that performance on tasks that are sensitive to ADHD is expected to correlate with continuous ADHD symptom scores in the general population. This has received scant attention in research to date, in contrast to a wealth of studies comparing task performance between children with ADHD and control children.

Amongst the most commonly used tasks in ADHD research are inhibition measures, such as the go/no-go and stop tasks. Whereas findings have been inconsistent with regard to a hypothesised inhibition deficit (see below), other data give further clues to the possible psychological processes implicated in ADHD. Two studies indicate an association between ADHD and poor inhibition on the stop task only in a standard, non-incentive condition, with the children with ADHD performing as well as the control children in a high incentive condition [10,11]. A third study did not find effects of reward and response cost on inhibition in ADHD [12], but the lack of comparison to a non-contingency condition limits the conclusions that can be made. Van der Meere and colleagues [13] found that inhibition on the go/no-go task was dependent on the presentation rate of stimuli: in contrast to both the slow and fast conditions, the performance of children with ADHD was comparable to that of control children in the medium event rate condition. In two further studies no differences were observed between ADHD and control groups on the inhibition indices of the stop task under two [14] or three [15] different event rates. Consistently across the studies, however, the slow condition produced disproportionately slow and variable reaction times in the children with ADHD. Event rate manipulation within the go/no-go task has also distinguished children with ADHD from control children with regard to cardiac [14] and evoked potential [16] measures: group differences, consistent with poor effort allocation in children with ADHD, emerged only in the slow and not in the fast condition.

Other recent studies similarly report the highly characteristic pattern on reaction time data, while not finding evidence for an association between ADHD and an inhibition deficit [7,17]. As Castellanos and Tannock [4] point out, "*response variability is the one ubiquitous finding in ADHD research across a variety of speeded-reaction-time tasks, laboratories and cultures*" (p. 624). A detailed statistical analysis of response times on a four-choice reaction time task demonstrated explicitly how a greater proportion of abnormally slow responses, mixed with fast responses on some trials, leads to the inconsistent pattern of responding in ADHD [18].

One approach to explaining the findings of highly variable cognitive performance and the interaction between cognitive performance and factors affecting the energetic state (incentives, presentation rate and predictability of stimuli) proposes an underlying regulation problem in ADHD. For example, the optimal stimulation theory placed the emphasis on the regulation of arousal [19] and, more recently, the state regulation theory proposes a critical role for the regulation of activation and effort (reviewed in [5]; see also [20]). Within this approach, the focus has also shifted from attentional *deficits *to the *regulation *of attention in ADHD [5,21,22]. An alternative account suggests that multiple psychological processes, both cognitive and reward-related processes, are affected [4].

Studying the psychological processes in ADHD within a genetic design is only at its beginning: candidate endophenotypes are being speculated upon and frameworks developed [4], but evidence is as yet limited. Initial studies have provided inconsistent findings, which has been attributed to methodological limitations, such as small sample sizes (reviewed in [1].

In this study we addressed two underlying assumptions that are highly relevant for large-scale genetic investigations on ADHD that include cognitive testing: (1) adequate test-retest reliability for key experimental tasks and (2) an association between performance on the tasks and ADHD symptom scores in a general population sample. We have previously reported test-retest reliability data for selected executive function measures and a 'delay aversion' task [23], and used these data to select tasks for a subsequent, initial twin study on hyperactivity [7,24]. The initial twin data supported reaction time variability as a potential endophenotype [24], with further supportive evidence emerging from a recent family study on ADHD [25]. In the current study we focused on two different tasks that appear particularly promising based on recent data – the go/no-go task and a reaction time task – and included within-task manipulations (some novel) with event rate and incentives. As working memory deficits are also proposed to characterise ADHD in some models [4], we additionally examine the association between ADHD symptom scores and performance on a working memory measure.

## Methods

Ethical approval was obtained from the Institute of Psychiatry Ethical Committee (Research).

### Sample

The children were recruited from local primary (ages 8–9 years) and secondary (ages 12–13 years) schools in London, UK. We asked head teachers to randomly select pupils who fulfilled the inclusion criteria of being fluent in English, within the appropriate age range and without special educational needs (severe sensory, physical or cognitive impairments). We specified that 'random selection' means a procedure such as choosing every fifth child in alphabetical order and that by using such random selection we aim to obtain a sample that is representative of children living in the UK. Consent forms and information packs were given to parents, who returned them to the school. Of the 66 parents contacted, 53 (80%) gave consent for their child to take part in the study. Each school that participated was given a £40 voucher to a book/stationery shop and the children earned small prizes (vouchers and stationery) on some tasks. Parents did not receive any financial reward for participation.

Children with prorated IQs below 70 (n = 3) were excluded. A fourth child was excluded due to a refusal to follow task instructions. The sample consists of 49 children: 19 boys and 30 girls. The children had a mean age of 11.03 years (SD = 2.08). The ethnic origin of the children was classified as follows: 51% white Caucasian, 35% African/Caribbean, 6% Indian/Pakistani/Bangladeshi, 2% Chinese, 4% 'other' and 2% unknown. Three children were not at school during re-test days and therefore time 2 data are missing for them. In addition, data are missing for a few children on specific tasks, due to technical problems with the portable computers during task administration (the numbers of children in each analysis are reported in the tables of the Results section).

### Procedure

Two trained research workers visited the children at school, assessing the children individually in separate, quiet rooms. The same research worker tested the same children at both time points. The test-retest interval was two weeks.

The tasks were administered in the following fixed order: fast task, WISC block design, WISC vocabulary, go/no-go task and WISC digit span. The tests, as well as conditions within each test, were presented in the same order at both time points. Testing sessions were arranged around the school's timetable and the children were given short breaks as required. The total length of the testing session, including breaks, was approximately 1.5–2 h.

### Measures

#### The Revised Conners' Parent [26] and Teacher [27] Rating Scales

Ratings on the Conners' scales were obtained from parents and teachers, with response rates of 98% and 83%, respectively. Adding up the scores on the inattentive and hyperactive-impulsive DSM-IV symptoms subscales forms a total ADHD DSM-IV symptoms subscale. A prorating procedure was applied in the few instances where there was missing data, as recommended in the manual [28].

#### Wechsler Intelligence Scales for Children (WISC-III^UK ^[29]

The vocabulary and block design subtests from the WISC were used to obtain an estimate of the child's IQ (prorated following procedures described by Sattler [30]). The children's IQs ranged from 72 to 145, normally distributed (M = 97.88, SD = 14.13). In addition, the digit span subtest was administered to obtain an estimate of working memory (digit span backward score).

#### The go/no-go task

We used a version of the task developed by van der Meere and colleagues [13,14]. On each trial, one of two possible stimuli appeared for 300 ms in the middle of the computer screen. The child was instructed to respond only to the 'go' stimuli and to react as quickly as possible, but to maintain a high level of accuracy. The proportion of 'go' stimuli to 'no-go' stimuli was 4:1. The response variables are commission errors, mean reaction time to 'go' stimuli and standard deviation of the reaction times. (Omission errors were rare – mean in each condition 1–5% – and are therefore not included in analyses.)

The children performed the task under three different conditions, matched for length of time on task. The fast condition consisted of 462 trials and had an inter-stimulus-interval (ISI) of 1 s. The ISI dropped to 8 s in the slow presentation condition, which consisted of 72 trials. The order of presentation of the slow and fast conditions was varied randomly across children.

The incentive condition was always administered last. This new condition, designed specifically for this study, is a modification of the incentive condition used in the study on the stop task by Slusarek et al. [11]. Each correct response to the letter X and each correct non-response to the letter O earned the child one point. The child lost one point for each omission error (failure to respond to X) and for each failure to respond within 2 s. Each commission error (incorrect response to O) led to the loss of five points. The points were shown in a box, immediately right of the screen centre, and were updated continuously throughout. The child started with 40 points, to avoid the possibility of a negative tally. The child was asked to try to win as many points as possible, and was told that the points will be exchanged for a real prize when the game ends. This condition consisted of 72 trials and had an ISI of 8 s.

A practice session preceded each experimental condition.

#### The fast task

The baseline condition followed a standard warned four-choice reaction time task, as outlined in Leth-Steensen et al. [18]. A warning signal (four empty circles, arranged side by side) first appeared on the screen. At the end of the foreperiod (presentation interval for the warning signal), the circle designated as the target signal for that trial was filled (coloured) in. The child was asked to make a compatible choice response by pressing the response key that directly corresponded in position to the location of the target stimulus. Following a response, the stimuli disappeared from the screen and a fixed inter-trial interval of 2500 ms followed. Speed and accuracy were emphasised equally. If the child did not respond within 10 s, the trial terminated.

First a practice session was administered, during which the child had to respond correctly to five consecutive trials. The baseline condition, with a foreperiod of 8 s and consisting of 72 trials, then followed.

To investigate the extent to which a response style characterised by slow and variable speed of responding can be maximally reduced, we developed a novel comparison condition that used a fast event rate (1 s) and incentives. This condition started immediately after the baseline condition and consisted of 80 trials (following the faster event rate conditions in Leth-Steensen et al. [18]). The child was told that if she will respond really quickly one after another, she will win smiley faces and will get real prizes in the end. The child won a smiley face each time she responded faster than her own mean reaction time during the baseline (first) condition consecutively for three trials. The baseline mean reaction time was calculated here based on the middle 94% of responses, therefore excluding extremely fast and extremely slow responses. The smiley faces appeared below the circles in the middle of the screen and were updated continuously.

Following the procedure recommended by Leth-Steensen et al. [18], observations on the fast task that were more than four standard deviations from a participant's mean reaction time for a specific condition were excluded. This conservative criterion aims to minimise the risk of removing any 'real' data in ADHD research on standard reaction time tasks, while still controlling for very extreme observations. Only 0.5–0.8% of the observations from each condition were excluded.

The response variables are mean reaction time and standard deviation of the reaction times, calculated for each condition based on correct responses only. (Trials that were therefore excluded consisted of omission errors (mean in each condition below 0.5%) and incorrect responses (mean in each condition 4–9%). We report data from the baseline condition both including all trials and including the first 30 trials only; the latter provides a match on length of time on task with the fast-incentive condition. Only the first 30 trials of the baseline condition were used to calculate difference scores, which indicate improvement between the baseline and fast-incentive conditions.

## Results

### Test-retest reliability

To establish test-retest reliability, we calculated inter-class Pearson product moment correlations and partial correlations, controlling for age, for each of the response variables (Table 1). Following the practice recommended by Rousson, Gasser and Seifert [31], we focus on inter-class rather than intra-class correlations (although report both to enable a comparison with previous research). Rousson et al. [31] recommend only using intra-class correlations, which take into account systematic error (learning effects), for intra-rater and inter-rater reliability, and not for test-retest reliability. Learning effects are a natural phenomenon and not a shortcoming associated with measures; in test-retest reliability the focus is on the consistency of the performance of the participants *in relation to *one another. To indicate the extent to which performance improved consistently across participants, we also report t-test results for the comparisons between mean scores at time 1 and time 2 for each measure (Table 1). For the test-retest correlations, where significance is not a sufficient criterion for adequate reliability, we focus on the size of the correlations. In further correlational analyses, we additionally indicate those correlations that reached significance at alpha .01 or .05 level (ie, if significance level is not given, this indicates a non-significant p-value).

**Table 1:** Test-retest reliability results

Measure	inter-class r	partial r^a^	intra-class r	time 1		time 2		t-value	df	p
								
				mean	SD	mean	SD			
**Fast Task (n = 41)**										
baseline (all trials)										
mean RT	.85	.75	.77	730.31	177.76	659.24	156.71	4.89	40	<.001
SD of RTs	.60	.53	.58	193.77	103.55	171.86	118.49	1.40	40	.17
baseline (30 trials)										
mean RT	.76	.53	.68	670.64	155.09	603.32	138.74	3.75	40	.001
SD of RTs	.47	.37	.43	155.78	68.90	141.59	97.80	0.93	40	.36
fast-incentive										
mean RT	.89	.79	.82	537.69	121.55	499.78	100.97	4.29	40	<.001
SD of RTs	.76	.65	.70	122.80	50.63	105.34	47.43	3.25	40	.002
**Go/No-Go (n = 44–47)**										
slow condition										
mean RT	.76	.63	.74	512.00	105.13	532.07	112.06	-1.77	44	.08
SD of RTs	.58	.53	.58	177.51	115.86	172.33	123.30	.315	44	.75
commission errors	.56	.56	.53	45.19	23.18	36.59	22.46	2.69	44	.01
fast condition										
mean RT	.88	.85	.87	387.20	74.69	385.24	64.58	0.37	44	.71
SD of RTs	.83	.82	.83	147.42	69.48	147.02	65.80	0.07	44	.95
commission errors	.70	.67	.64	42.54	16.99	38.62	17.89	1.93	44	.06
incentive condition										
mean RT	.70	.63	.69	508.64	91.44	526.48	79.26	-1.77	43	.08
SD of RTs	.26^b^	.09^b^	.26	119.52	47.43	122.91	40.47	-0.42	43	.68
commission errors	.54	.47	.52	25.95	16.30	21.82	16.97	1.72	43	.09

^a ^controlling for age ^b ^inter-class r increases to .61 and partial r to .44, if exclude six potential outliers (see text for further information)

The test-retest correlations for both tasks were in the moderate to high range (.5–.9), with two exceptions (see below). The results were overall similar whether or not age was controlled for, with only slight decreases in the size of the correlation for the partial correlations. Significant t-test results, indicating learning effects, emerged for all other fast task variables, except the standard deviation of reaction times in the baseline condition, but for only one go/no-go task variable (commission errors in the slow condition).

For the baseline fast task data there was a small decrease in the size of the test-retest correlations (from .5–.9 to .4–.8), when considering the first 30 trials separately. However, the fast-incentive condition data indicated good reliability, despite the relatively short administration time.

The low inter-class correlation of .26 for the standard deviation of reaction times in the incentive condition of the go/no-go task increased to .61, and the partial correlation to .44, if six children with highly discrepant values between time 1 and time 2 sessions (differences of 92–158 ms) were excluded. To examine the extent to which the lower reliability of this variable may affect its validity, insofar as its association with theoretically related variables is concerned, we calculated a Pearson product moment correlation between this variable (including the potential outliers) and the standard deviation of reaction times in the fast-incentive condition of the fast task. For time 1 data the correlation was .56 (p < .01); for time 2 data the correlation dropped to .29.

### Effects of task manipulations on the total sample

Paired t-tests between each pair of comparison conditions within a task indicated improved performance (p < .01) following task manipulations (fast event rate and/or incentives) for all but three go/no-go task comparisons: (1) SD of RTs between slow and fast conditions, (2) commission errors between slow and fast conditions and (3) mean RT between slow and incentive conditions. To minimise possible practice effects (given that the incentive conditions were always administered last), we performed these analyses on time 2 data.

To explore the extent to which the two types of manipulations in the go/no-go task – fast event rate and incentives – had a similar effect on performance within individual children, we calculated Pearson product moment correlations, and partial correlations controlling for age, between the two difference scores for each variable. In these as well as other correlational analyses we present both types of correlations to allow for an easy visualisation of the effects of age on the results. However, as the main interest is on associations between performance across conditions (or between task performance and ADHD ratings, below) *independent of age effects*, we focus mainly on the partial correlations. The slow-fast condition difference score and the slow-incentive condition difference score correlated strongly for each of the three variables (all p < .01): mean RT (r = .63/partial r = .62), SD of RTs (r = .78/partial r = .77) and commission errors (r = .73/partial r = .74).

### Association between task performance and ratings of ADHD symptoms

The next question we addressed was whether individual differences between children on task performance and, in particular, the extent of improvement from the slow baseline condition to a condition with a faster event rate or incentives (or both) is associated with parents' and teachers' ratings of ADHD symptoms (T-scores). The model of arousal and activation regulation predicts an association between ADHD symptoms and poor performance in a baseline condition, but lack of (or reduced) association with performance in conditions with such task manipulations. Here we focus on time 1 data only, which reflect the usual situation of a one-off assessment session. We calculated Pearson product moment correlations, and partial correlations controlling for age, between these variables and report also the associated effect sizes (Tables 2, 3, 4).

**Table 2:** Association between difference scores on the go/no-go task and parent and teacher ratings on the Conners' scales (T-scores): correlations (*r*) and partial correlations, controlling for age (*partial r*), with associated effect sizes (*d*)

Difference score	Teacher (n = 38–39): total ADHD symptom score				Parent (n = 45–46): total ADHD symptom score			
	*r*	*d*	*partial r*	*d*	*r*	*d*	*partial r*	*d*
slow – fast condition								
mean RT	.19	0.4	.24	0.5	-.11	-0.2	-.06	-0.1
SD of RTs	.44**	1.0	.44**	1.0	.08	0.2	.13	0.3
commission errors	.39*	0.8	.40*	0.9	.11	0.2	.12	0.2
slow – incentive condition								
mean RT	.36*	0.8	.38*	0.8	.15	0.3	.19	0.4
SD of RTs	.44**	1.0	.46**	1.0	.13	0.3	.17	0.3
commission errors	.21	0.4	.20	0.4	-.03	-0.1	-.06	-.01

* p < .05 (2-tailed); ** p < .01 (2-tailed)

**Table 3:** Association between go/no-go task variables and parent and teacher ratings on the Conners' scales (T-scores): correlations (*r*) and partial correlations, controlling for age (*partial r*), with associated effect sizes (*d*)

Measure	Teacher (n = 38–39): total ADHD symptom score				Parent (n = 45–46): total ADHD symptom score			
	*r*	*d*	*partial r*	*d*	*r*	*d*	*partial r*	*d*
slow condition								
mean RT	.10	0.2	.22	0.5	-.19	-0.4	-.09	-0.2
SD of RTs	.39*	0.8	.48**	1.1	.05	0.1	.14	0.3
commission errors	.38*	0.8	.42**	0.9	.02	0.0	.06	0.1
fast condition								
mean RT	-.08	-0.2	-.01	-0.0	-.15	-0.3	-.04	-0.1
SD of RTs	-.06	-0.1	-.03	-0.1	-.05	-0.1	-.01	-0.0
commission errors	.10	0.2	.14	0.3	-.10	-0.2	-.05	-0.1
incentive condition								
mean RT	-.26	-0.6	-.23	-0.5	-.35*	-0.7	-.29	-0.6
SD of RTs	-.09	-0.2	-.03	-0.1	-.21	-0.4	-.12	-0.2
commission errors	.21	0.4	.31	0.7	.02	0.0	.13	0.3

* p < .05 (2-tailed); ** p < .01 (2-tailed)

**Table 4:** Association between fast task variables and parent and teacher ratings on the Conners' scales (T-scores): correlations (*r*) and partial correlations, controlling for age (*partial r*), with associated effect sizes (*d*)

Measure	Teacher (n = 36): total ADHD symptom score				Parent (n = 43): total ADHD symptom score			
	*r*	*d*	*partial r*	*d*	*r*	*d*	*partial r*	*d*
baseline (all trials)								
mean RT	.19	0.4	.42**	0.9	-.06	-0.1	.13	0.3
SD of RTs	.45**	1.0	.58**	1.4	.16	0.3	.29	0.6
baseline (30 trials)								
mean RT	.14	0.3	.35*	0.7	-.11	-0.2	.06	0.1
SD of RTs	.44**	1.0	.55**	1.3	.17	0.4	.28	0.6
fast-incentive								
mean RT	-.03	-0.1	.10	0.2	-.08	-0.2	.10	0.2
SD of RTs	.09	0.2	.22	0.5	.08	0.2	.26	0.5
difference score (baseline 30 trials – fast-incentive)								
mean RT	.20	0.4	.24	0.5	.07	0.1	-.03	0.0
SD of RTs	.47**	1.1	.47**	1.1	.14	0.3	.14	0.3

* p < .05 (2-tailed); ** p < .01 (2-tailed)

For the go/no-go task the teacher-rated ADHD symptoms correlated moderately strongly (.4–.5) and significantly with four out of the six difference scores (Table 2), indicating a greater improvement in performance between the slow and the other two conditions in children with more ADHD symptoms. An additional examination of the correlations from the individual conditions confirmed this pattern of findings (Table 3): moderately strong correlations (.4–.5) were obtained for performance in the slow condition, and low correlations – indeed in some cases negative correlations, indicating better performance – were obtained for performance in the fast and incentive conditions. The correlation between teacher-rated ADHD symptoms and the difference score for commission errors between the slow and incentive conditions was less strong (.2) and non-significant. In contrast, no clear pattern emerged for correlations with parent-rated ADHD symptoms.

For the fast task the teacher ratings were moderately strongly (.5) associated with the standard deviation of reaction times difference score, indicating an association between teacher-rated ADHD symptoms and improvement in the variability in the speed of responding from the baseline to the fast-incentive condition (Table 4). This was also reflected in the highly significant correlations with baseline data, and the lower and non-significant correlations with fast-incentive condition data. Correlations with parent ratings were all non-significant and do not indicate a clear pattern.

We calculated additional correlations, controlling for age, separately for the inattentiveness and hyperactivity-impulsivity subscales for the key measures on both tasks (the difference scores), to examine whether a similar pattern of results emerges for both subscales. These suggested few differences for either teacher or parent ratings. The magnitude of the difference between the correlation of a difference score with inattentiveness *vs *hyperactivity-impulsivity was less than .12 in each case, with neither dimension consistently associated with higher correlations.

The digit span backward score, a measure of working memory, was not associated with either teacher (r = .09) or parent (r = .04) ratings of ADHD symptoms. The correlations with digit span forward scores were similarly near zero (r = -.03 and r = .01, respectively).

As a greater proportion of teacher ratings were missing for boys in the older age range, than for the other subgroups of children, we additionally examined correlations between task performance and parent ratings, excluding children for whom teacher ratings were missing. The correlations indicated a similar pattern of results as with the total sample.

The teacher and parent inattentiveness ratings correlated .28 with one another, and the teacher and parent hyperactivity-impulsivity ratings .33. For the total ADHD symptoms, the correlation between teacher and parent ratings was .33 (p < .05).

The association between IQ and task performance is not a specific focus of this paper, but we note that the pattern of associations between the key task variables (difference scores) and parent and teacher ratings of ADHD symptoms did not change, when controlling for IQ, as well as for age.

## Discussion

With a general population sample of children, we demonstrated moderate to good test-retest reliability for the majority of variables from the go/no-go and fast tasks, and an association between performance on these tasks and teacher, but not parent, ratings of ADHD symptoms.

An acceptable level of test-retest reliability depends on the nature of the measures. For tests to be useful in clinical practice, high reliability coefficients are commonly required (around .8 or above [33]). With experimental tasks, it may be unrealistic to expect reliability coefficients of such magnitude. We previously reported test-retest inter-class correlation coefficients of between .2 and .7 (with a median value of .66) for a range of task variables [23].

Considering correlations of .7 or higher as indicating good test-retest reliability, and correlations of .5 and .6 as indicating moderate test-retest reliability, the variables from the fast and go/no-go tasks were within such a moderate-to-good reliability range, with two exceptions. Controlling for age effects did not have a noticeable effect on the reliability coefficients, with only slight decreases in the size of the correlation for some variables.

Reaction time variability in the baseline condition of the fast task indicated adequate test-retest reliability when including all trials, but the inter-class and partial correlation coefficients were slightly lower (.5 and .4) when focusing on the first 30 trials only. This illustrates how task length can affect reliability. The lower reliability coefficient for the reaction time variability in the incentive condition of the go/no-go task seems due to a few children having highly discrepant values between the test and retest sessions. The reason for this is not clear, but we note that the incentive condition was administered late in the testing session and this may have contributed to fatigue in some children. It seems relevant that the reaction time variability in the go/no-go task incentive condition was strongly associated with the reaction time variability in the fast-incentive condition of the fast task at time 1, as predicted, but that the association was less strong (and non-significant) at time 2. Given that these tasks are aimed to challenge children's ability to concentrate over time, fatigue may have affected some children's performance particularly at time 2 (when novelty of the tasks had worn off). Note that in the other two conditions the reaction time variability demonstrated adequate test-retest reliability.

When using these tasks within genetic designs, we aim to use multivariate genetic model-fitting approaches. From a test-retest reliability viewpoint such analyses may have an additional advantage: *a priori *one would expect a better reliability for summed components compared to single variables [31].

The second main research question addressed the extent of association between task performance and ratings of ADHD symptoms in a general population sample. The within-task manipulations with event rate and incentives test the prediction of the arousal and activation regulation model of improved performance following such manipulations; therefore associations are predicted with difference scores, which indicate improvement from 'baseline' to a comparison condition. When considering the data separately from each condition, this would be reflected in associations with baseline performance and lack of (or reduced) associations with performance in the conditions with incentive or event rate manipulations.

The associations with teacher ratings of ADHD symptoms were overall in line with these predictions. The strongest associations were obtained for reaction time variability. In both tasks the decrease in reaction time variability from baseline condition to a condition with a faster event rate or incentives (or both) was significantly associated with teacher ratings of ADHD symptoms. The data from each condition separately indicate how the difference score results reflect the significant associations of teacher ADHD ratings with reaction time variability in the baseline conditions, and low or no association with this variable in the conditions with event rate and incentive manipulations. The magnitude of the correlations (.4 – .6) is greater than, for example, that between ADHD symptoms and IQ scores, which is typically -.2 – -.4 [32,34-36]. (Note that a correlation of -.3 translates to a 9 point difference in mean IQ scores between children with ADHD and control children [32]). In the fast task the association with teacher ADHD ratings emerged in the baseline condition whether focusing on the first 30 trials only or the full 72 trials. Similar results, though somewhat less strong, emerged also for mean reaction times. The association between teacher-rated ADHD symptoms and disproportionately slow and variable reaction times in the slow condition is consistent with previous studies on children with ADHD [13-15]. In addition to our focus on individual differences in relation to ADHD symptoms, we also demonstrated that the fast task manipulations improved the speed and the variability of speed in the total sample of children.

On the go/no-go task, the rate of commission errors is considered indicative of response inhibition. Teacher ratings of ADHD symptoms were associated with commission errors in the slow but not in the fast condition. Findings on the effects of event rate on inhibition in children with ADHD have been somewhat inconsistent, with uncertainty surrounding an optimal event rate and some studies reporting no association between commission errors and ADHD in any event rate condition [14,15]. The present data on associations with teacher ratings of ADHD symptoms are consistent with the suggestion that a fast event rate optimises the activation or arousal state of the child [15]. The findings were less strong regarding improvement in the rate of commission errors from the slow to the incentive condition: although in the predicted direction (r = .2), the correlation between teacher ratings of ADHD symptoms and the difference score was not significant. In our future research we will investigate the effects of the incentive manipulation in the go/no-go task in children with diagnosed ADHD.

The data on the go/no-go task and teacher (or parent) ratings of ADHD symptoms are not consistent with an inhibition deficit hypothesis that predicts an association between ADHD symptoms and commission errors across all conditions. We similarly obtained no evidence for an association between ADHD symptom scores and working memory, as measured by the digit span backward score. The present data demonstrated effects of event rate and incentives and further indicated that the two types of manipulations in the go/no-go task had a similar effect on performance within individual children: improvement in inhibition and reaction time performance following a faster event rate correlated strongly with improvement in performance following the introduction of incentives. Whereas effects of individual manipulations may be open to multiple interpretations, the overall pattern of findings obtained in the present study, including the association between the effects of the two types of manipulations in the go/no-go task, seems most parsimonious with the model of arousal and activation regulation. Yet the possible relationships between different current models need to be investigated in more detail.

In contrast to the encouraging findings with teacher ratings, no clear pattern of findings emerged for parent ratings of ADHD symptoms. Oosterlaan and colleagues [37] similarly reported recently that only teacher ratings of ADHD symptoms predicted performance on cognitive tasks that were sensitive to ADHD, with parent ratings not contributing to the association. The sample in their study included children with research diagnoses of pervasive ADHD and control children.

Teacher and parent ratings of ADHD symptoms reflect only partially overlapping phenotypes (as exemplified by the correlation of .3 reported here) and genotypes [38]. Potential strengths of teacher ratings of ADHD symptoms include a better awareness of population norms, observing children in situations that are challenging for children with ADHD symptomatology and greater objectivity. Teacher ratings show higher internal consistency and stability [39], are free of the rater bias typically found in parent ratings [40] and show also greater genetic stability [41]. However, the validity of teacher and parent ratings is a complex issue and a detailed discussion is beyond the scope of this paper.

An exploration of the degree of association of inattentiveness and hyperactivity-impulsivity subscales separately with key task variables suggested few differences. Data from studies on diagnosed ADHD on the strength of the association between each subtype and task performance have not yielded a consistent pattern [10,37,42,43].

A limitation of the current study is that a greater proportion of teacher ratings were missing for boys in the older age range, than for the other subgroups of children. However, the response rate from teachers was high overall (83%) and we controlled for age effects in the analyses. Additional analyses also indicated that the missing teacher ratings are unlikely to have led to any systematic bias. Another limitation, though not a specific focus of the study, is that the sample was not large enough to analyse data separately for girls and boys to study possible sex effects. Although ADHD diagnoses are more common among boys than girls [44], from a QTL perspective the focus is not only on diagnosed ADHD, but also on ADHD symptoms in an unselected population; hence an interest in studying the reliability and validity of the tasks in a mixed-sex sample.

## Conclusion

The demonstration of moderate-to-good test-retest reliability for the majority of the variables from the go/no-go and fast tasks supports their usefulness for genetic studies, which require reliably measured indices of individual differences. The association of task performance (including variables previously associated with clinically diagnosed ADHD) with teacher ratings of ADHD symptoms in a general population sample is consistent with the assumption of a continuously distributed ADHD trait. The lack of a clear pattern of findings with parent ratings of ADHD symptoms warrants further study. The next step will involve an examination of the QTL hypothesis of a quantitative dimension of ADHD symptoms in endophenotype research, by using the same tasks in genetic designs with a general population sample with continuously measured ADHD symptoms, as well as with children with diagnosed ADHD. Combining quantitative genetic (family or twin designs) and molecular genetic methods will enable an investigation of both the overall familial or genetic influences on task performance and associations between specific genes and task performance.

## Competing interests

The author(s) declare that they have no competing interests.

## Authors' contributions

JK conceived of the study, supervised task development, recruitment and data collection, performed statistical analyses and drafted the manuscript. PA participated in recruitment, task development and data collection, and performed statistical analyses on data on the go/no-go task. JM participated in recruitment, task development and data collection. NAB and JJvdM developed the go/no-go task and advised on statistical analyses on this task. All authors read and approved the manuscript.

## Pre-publication history

The pre-publication history for this paper can be accessed here:


